# Recyclability of Post-Consumer Polystyrene at Pilot Scale: Comparison of Mechanical and Solvent-Based Recycling Approaches

**DOI:** 10.3390/polym15244714

**Published:** 2023-12-15

**Authors:** Jean-Mathieu Pin, Iman Soltani, Keny Negrier, Patrick C. Lee

**Affiliations:** 1 Polystyvert Inc., 9350 Rue de l’Innovation, Anjou, QC H1J 2X9, Canada; 2 Multifunctional Composites Manufacturing Laboratory (MCML), Department of Mechanical and Industrial Engineering, University of Toronto, 5 King’s College Road, Toronto, ON M5S 3G8, Canada

**Keywords:** polystyrene recycling, solvent-base recycling, decontamination, mechanical recycling, post-consumer feedstock

## Abstract

Solvent-based and mechanical recycling technology approaches were compared with respect to each process’s decontamination efficiency. Herein, post-consumer polystyrene (PS) feedstock was recycled by both technologies, yielding recycled PS resins (rPS). The process feedstock was subjected to four recycling cycles in succession to assess the technology perennity. The physico-chemical and mechanical properties of the rPS were then evaluated to discern the advantages and drawbacks of each recycling approach. The molecular weight of the mechanically recycled resin was found to decrease by 30% over the reprocessing cycles. In contrast, the solvent-base recycling technology yielded a similar molecular weight regarding the feedstock. This consistency in the rPS product is critical for consumer applications. Further qualitative and quantitative analyses on residual organic compounds and inorganic and particulate contaminants were investigated. It was found that the solvent-based technology is very efficient for purifying deeply contaminated feedstock in comparison to mechanical recycling, which is limited to well-cleaned and niche feedstocks.

## 1. Introduction

Sustainability in the plastic industry has become increasingly important as existing technologies evolve to comply with circular economy requirements. Owing to the breadth of their applications, the increase in plastic production and usage is predicted to continue, with the 367 Mt of plastic produced in 2020 doubling by 2040. Unfortunately, the current linear economy generates a staggering amount of waste, with about 5 Bt released into the environment since the advent of large-scale plastic production [1,2,3].

Generally, most plastic waste can be categorized as macro-, micro-, or nano-plastics. While macro-plastic waste has harmful effects on wildlife, micro- and nano-plastic waste is a potential environmental threat depending on particle shape, size, and polymer composition, as well as the presence of additives, often imparting long-lasting effects when ingested [2,4]. However, with increasing environmental awareness regarding the consequences of plastic pollution, as well as the socio-economic benefits boasted by waste re-valorization, governments are actively pushing to promote a transition from the traditional linear economy to a circular economy on an international scale. The implementation of a circular economy means the re-construction of entire material life cycles from material production to consumer use, followed by end-of-life management [5]. Nevertheless, the closing of this open loop starts with feedstock waste handling [6].

Plastic waste feedstocks can be primarily classified under two categories: post-industrial and post-consumer feedstocks. Post-industrial feedstock refers to residual materials obtained from production lines or from manufactured goods that do not meet quality control standards. When possible, these materials are re-introduced into extrusion lines and are repurposed. Unfortunately, this is often challenging to implement. As an example, a multilayer composite cannot be reprocessed mechanically because of its intrinsic anisotropy. On the other hand, post-consumer feedstock refers to plastic material waste obtained after consumer use. Typically, post-consumer feedstocks are heavily contaminated with a myriad of contaminants such as dust, oil and grease, adhesives, etc. Moreover, while plastics are typically sorted by their density and chemical structure [7], sorting processes are not completely efficient, yielding cross-contamination with other type of plastics. Due to this complexity of contamination and the lack of industrial efficient technology of recycling, the life cycle of PS currently ends with accumulation in landfills or the environment.

Different recycling methods exist for PS at different stages of maturity and use [8]. Multiple examples from academia propose upcycling strategies, repurposing PS in added value materials [9,10,11]. However, nowadays, only mechanical recycling is used for commercial application, to the best of our knowledge. Perpetual optimization of the technology by adding washing pre-treatments of the feedstock and additives into the formulation led to several successes, permitting the number of times the same resins can be re-used to increase [12].

**Figure 1:**
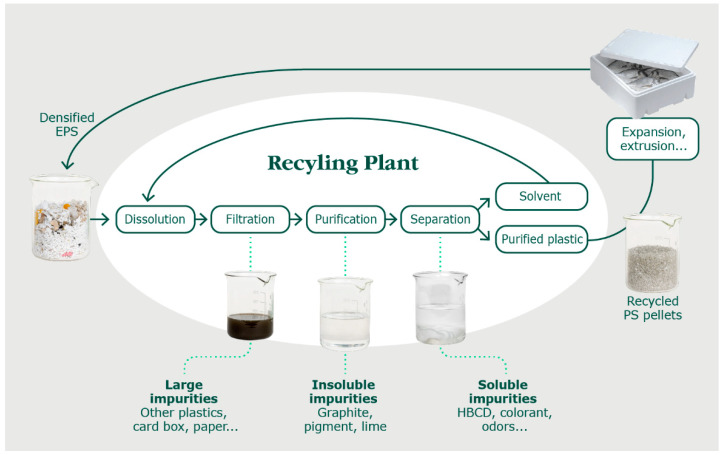
Polystyvert Inc.’s patented recycling process [10].

Some of the most suitable technologies in terms of recycled resin purity, cost, and sustainability are solvent-based technologies. To precisely assess the sustainability and cost of the technology, a thorough life cycle and economic analysis is required. However, the following reference gives a first insight on GHG emission, comparing several plastic end-of-life management technologies [13]. Herein, we describe a newly developed and patented solvent-based recycling technology for styrenic polymers, as is depicted in Figure 1 [14]. The feedstock is firstly mixed with the solvent *p*-cymene to selectively dissolve the PS. The *p*-cymene is a green solvent compared to its phenylic counterparts, such as toluene, owing to its low toxicity and high flash point (47 °C) [15]. The resulting solution is filtered to remove macro-contaminants such as adhesives, labels, other plastics, etc. Then, the smaller insoluble particles are removed using a 100 µm pore filter, followed by centrifugation, yielding a purified solution thereafter. In the next step, the PS is precipitated by adding heptane, the non-solvent, at its boiling point temperature (i.e., 98 °C at 1 atm) [16]. This step permits the recovery of the PS but also removes the soluble contaminants including colorants, oil/greases, and halogenated flame retardants such as hexabromocyclododecane (HBCD). HBCD removal is critical as this flame retardant has been banned in most countries due to its toxicity [14,17]. HBCD was used abundantly during the second half of the 20th century and is therefore currently still present in post-consumer feedstocks. Abdallah et al. pointed out an important issue when analyzing mechanically recycled PS in the UK. Feedstock containing HBCD was used for recycling and, therefore, ended up in new materials made with this recycled resin [18]. The PS precipitated paste is then washed with heptane, at its boiling point temperature, to maximize removal of soluble contaminants. The resulting washed paste enters into an extruder with a devolatilization system to remove the remaining solvents. This permits the production of recycled pellets that can be used in the same way as the virgin polymer. It is important to note that, in the industrial process, the solvents are recovered at several stages for a total yield of ≥98%, to be recycled in a distillation column for reuse.

This investigation aims to compare the recycling treatment efficiency of an EPS post-consumer feedstock by means of mechanical recycling and a non-destructive solvent-based technology over the course of four recycling process treatments. It is important to note that the feedstock is taken as is, without pre-treatment. Also, no additives are added for the mechanical recycling. The technology used in this study is the most basic approach developed by Polystyvert Inc. and is presented in detail in the following reference [14]. It should be noted that this procedure is replicated without using any of the deep purification modules presented in other references [17,19]. The recycled resin properties following several reprocessing cycles are of utmost importance in assessing the quality of the PS recovered from each recycling technology. Critical properties including the molecular weight and halogen content must be met from processing and commercial standpoints, to abide by quality control and government-imposed standards. In the following investigation, the sample nomenclature will be “SR-number” for solvent-based recycled PS and “MR-number” for mechanically recycled PS.

## 2. Materials and Methods

Materials: Post-consumer white expanded polystyrene (EPS) was used as the starting material for the recycling process; the feedstock used in both recycling processes came from the same source. As a post-consumer material, the EPS contained a variety of contaminants such as dust, tape, and organic and inorganic compounds, as well as expansion gas, additives, and fillers.

Chemicals: *p*-cymene (Haihang Industry, Jinan, China) was used to dissolve the EPS. This solvent is a colorless, clear liquid, recycled by distillation in house, as illustrated in Figure 1. The distilled *p*-cymene exhibited a purity of ≥99%. Heptane (Brenntag Canada Inc., Langley Twp, BC, Canada, technical grade) was used as the non-solvent. This solvent is a colorless, clear liquid, recycled in house, with a purity of 99.5%. Tetrahydrofuran (purity ≥ 99.9%), dichloromethane (purity ≥ 99.8%), toluene (purity ≥ 99.9%), cyclohexane (purity ≥ 99.8%), and polystyrene (Mw 192,000 g/mol) were purchased from Sigma-Aldrich, Oakville, ON, Canada.

Gel Permeation Chromatography (GPC): A Viscotek TDA302 GPCmax was used with two TSKgel GMHHR-M columns and a Refractive Index (RI) as detector. Prior to the analysis, a standard PS calibration curve was achieved with r^2^ = 0.999607. The pellet samples were left to dissolve under stirring in THF at room temperature overnight. The solutions were filtered through a 0.22 μm PTFE syringe filter. The filtered solutions were then injected at 30 °C with a flow rate of 0.7 mL/min. OmniSEC 4.6.2 software was used to analyze the triplicate results.

Gas chromatography coupled with mass spectrometry (GC-MS): The samples were injected into a GC Trace 1300 (SSL) coupled with an ISQ 7000 Mass Spectrometer detector (ThermoFisher Scientific, Waltham, MA, USA). A TG-5SILMS GC Column of 60 m × 0.25 mm was used to separate the analytes. Data recording and treatment were conducted using Chromeleon 7.2.1 software. The samples were prepared by adding the pellets of interest in dichloromethane, followed by sonication. After sonication for 10 min at RT, the polystyrene dissolved fully, and the analytes were thereby extracted. The solution was then filtered through a 0.45 μm PTFE microporous membrane prior to injection. Triplicates were performed on each sample.

UV–Vis spectroscopy: Samples were analyzed on an Agilent Cary 60 UV-Vis coupled with a standard 10 mm probe accessory. Measurements were conducted in transmission mode at a wavelength of 550 nm. Sample solutions were prepared at 10 wt.% PS in toluene. The zero was obtained using a sample made with virgin PS at the same polymer concentration. This was used as evidence of residual particles in the recycled resins.

Rheology: Rheological analysis of samples was performed using a strain-controlled ARES G2 rheometer equipped with 25 mm parallel plates and a gap of about 1 mm. Dynamic frequency sweeps were also employed within the linear viscoelastic region of the materials, at an oscillation strain of about 5%, to measure the complex viscosity (η*) as a function of frequency using the angular frequency range of 0.1 rad/s to 300 rad/s.

Tensile test: The tensile tests were conducted in accordance with ASTM D638 [20] on compression-molded Type V test pieces using an Instron universal machine model 5965. The tests were performed using an initial gauge length of 25 mm and a crosshead speed of 5 mm.min^−1^. Five test specimens were evaluated for each sample. The samples were aged at room temperature for ten days after being molded and prior to conducting the tensile test.

X-ray fluorescence (XRF) spectroscopy: A Bruker S1 Titan 800 was used coupled to a benchtop accessory. Measurement was performed in triplicate on each sample, and the average was calculated. The analysis was settled at 60 s: 30 s at 50 kV and 30 s at 15 kV.

Melt Flow Index (MFI): An Ametek davenport MFI 10 was used to measure the Melt Flow Index of the recycled pellets. The method was based on ASTM D1238 [21], and the following parameters were applied for the polystyrene: 200 °C/5.0 kg.

Methods for non-soluble particle quantification in recycled pellets: A 100 g amount of polymeric solution at 15 wt.% PS in toluene was prepared. This solution was subject to centrifugation at 8500 rpm for 20 min, with the supernatant separated from the tubes. The solid residues were recovered and washed thoroughly with toluene, firstly, and cyclohexane, secondly, to remove all the polymer from the particulate contaminants. The resulting solid residues were dried at 90 °C in a vacuum oven for 4 h. The dried residues were then weighed on a Mettler Toledo analytical scale (±0.0001 g) to calculate the % of non-soluble particles.

Mechanical recycling: The thermomechanical recycling of the PS was conducted using a single screw extruder with an aspect ratio of 24. Therefore, samples MR-1 to MR-4 were made by performing the extrusion on the PS for 4 repetitive cycles at 210 °C and with a residence time of about 35–50 s.

**Figure 2:**
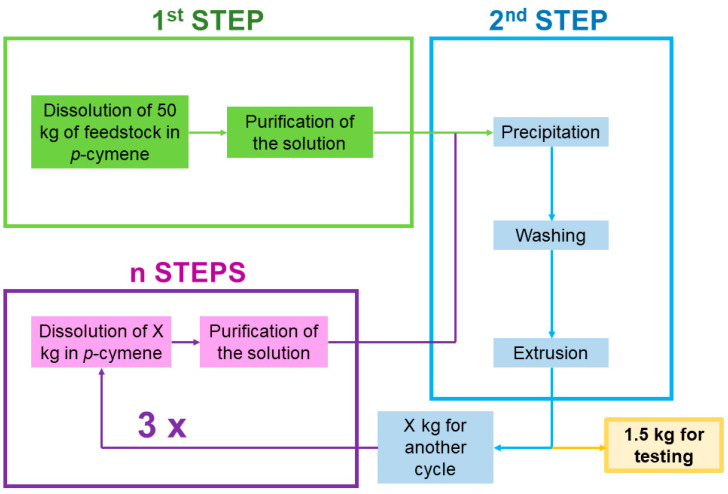
Solvent-based recycling procedure of PS resin used in the Polystyvert Inc. pilot plant.

Solvent-base recycling: As illustrated in Figure 2, the production of rPS samples was achieved at pilot plant scale following the protocol described below. The Polystyvert Inc. (Anjou, QC, Canada) pilot plant can produce 5 kg/h of recycled PS resin. This process can be divided in different modules. The first step is a selective dissolution of the PS-containing feedstock into *p*-cymene. After the first filtration stage (i.e., 100 µm filtration), the solution is further decontaminated from small particles by means of centrifugation. The PS is then selectively precipitated from this purified solution using heptane as a non-solvent. The obtained “paste” is then washed with heptane to remove the soluble contaminants. The resulting washed paste is transformed into pellets using a single screw extruder with one port of devolatilization. The parameters of extrusion were tuned to match the mechanical recycling extrusion parameters describe above. From the total weight of recycled PS produced in this first run, named SR-1, 1.5 kg was withdrawn for characterization. The remainder of the SR-1 pellets were dissolved again for the subsequent recycling cycle. Following the same steps as described above, SR-2 pellets were produced. Similarly, taking SR-2 pellets, SR-3 pellets were produced, and SR-4 pellets from SR-3 pellets.

## 3. Results and Discussion

**Figure 3:**
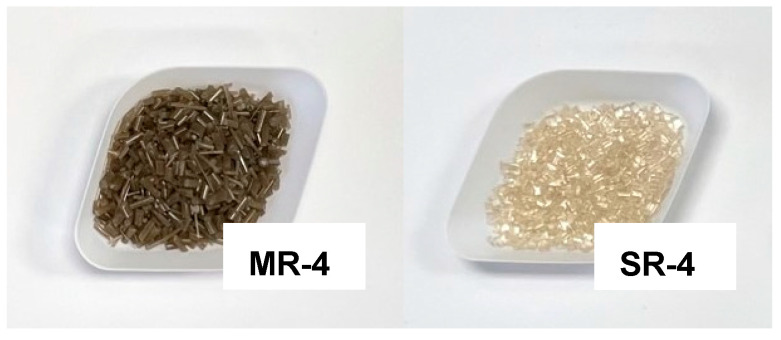
Generated recycled pellets obtained after the 4th reprocessing cycle.

Figure 3 illustrates resulting pellets from the MR and SR processes after four cycles of recycling. The MR-4 pellets are opaque and dark brown, while the SR-4 pellets are translucid and yellowish. The stark differences in color are due to several different factors. For the MR-processed samples, brown colors can be associated with extensive degradation of the polymer chains, which generate chromophores, as is characteristic of mechanical recycling approaches. In this case, most of the residual organic contaminants undergo degradation with the elevated temperature and shear stresses in the extrusion process. Typical biological contaminants, such as proteins, start to degrade at low temperatures and could contribute to this pigmentation. Moreover, the yellowish color from SR-4 could be ascribed to chromophores that are sporadically present along the polymer chains as a consequence of chemical aging induced by oxygen and light, as well as temperature [22].

In addition to the macroscopic observations illustrated in Figure 3, more quantitative information is presented in Table 1. To estimate the opacity of the recycled resin, UV–Vis measurements at 550 nm were conducted on the resin solutions. This clearly demonstrated that, in the case of SR-4, almost all light could be transmitted, while, for the MR-4, because of abundant suspended particulate matter, only half of the light was transmitted. These results are in good agreement with the measured weight of the remaining non-soluble contaminants, also presented in Table 1.

**Figure 4:**
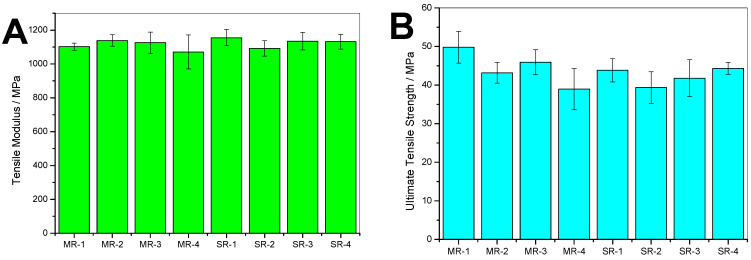

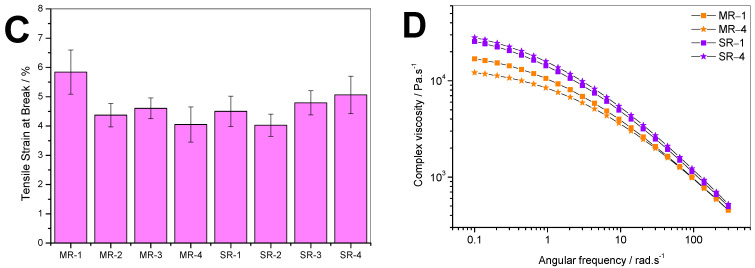
Tensile test measurements conducted on the recycled resins illustrating: (**A**) modulus, (**B**) tensile strength, and (**C**) elongation at break. (**D**) illustrates the complex viscosity measured by small amplitude oscillatory shear rheology.

The tensile properties exhibited in Figure 4A–C are typical of what is expected from PS resins. There were no significant differences in mechanical properties when comparing the MR with the SR resins. This is in good agreement with Remili et al. [23], who claimed that, while observing a significant change of the molecular weight, mechanical property decrement only becomes significant after the sixth reprocessing cycle. However, the complex viscosity exhibited in Figure 4D highlights significant changes. First, overall, the SR samples had higher viscosity than MR samples. Second, between MR-1 and MR-4, the viscosity was reduced by about 5000 Pa.s^−1^. This implies that PS is degraded during the mechanical recycling process, resulting in shorter chains that translate to a reduced melt viscosity [23]. The particulate and molecular contaminants could also have played a role in reducing the viscosity in the MR samples since no purification processes were applied. In contrast, the viscosity of SR samples was very close. Interestingly, the SR-4 sample’s viscosity was slightly higher than that of SR-1. To confirm this first observation, other approaches including MFI measurements and molecular weight quantification were performed.

**Figure 5:**
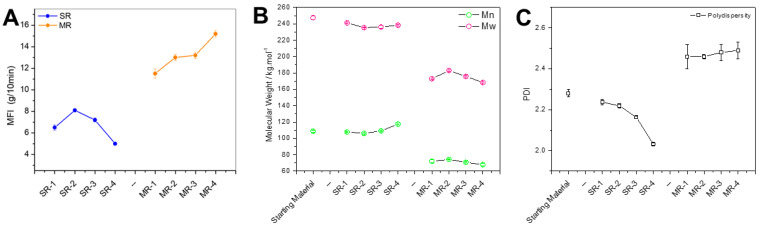
(**A**) MFI values for recycled resins after each reprocessing cycle; (**B**) molecular weight comparison between mechanical and solvent-based recycled resins and (**C**) associated polydispersity.

The MFI is one of the most important characteristics for industrial material processing purposes. Figure 5A clearly shows an opposite trend when comparing MR with SR resins. For mechanical recycling, the MFI increases with increasing number of reprocessing cycles. Conversely, the MFI of SR pellets was quite stable, with a slight reduction with increasing reprocessing cycles. The lowest recorded MFI was for the last cycle of the solvent-based recycling, with a value of 5.2 g/10 min. The typical factors that may affect the MFI are the resin molecular weight, residual solvents, additives such as lubricants, and particle contaminants. The trend observed for the complex viscosity is overall confirmed here with the intermediate reprocessing cycles. It is important to note that the degradation of PS results in *β*-scission of the polymer chain, leading to smaller chains, and a reduction in the viscosity. However, the generation of radicals could induce cross-linking that may increase the viscosity. While these two reactions are in competition, the literature suggests that *β*-scission becomes more preponderant with degradation completion [23].

To further comprehend the preponderant factors affecting the MFI, the molecular weight of the recycled resin was monitored throughout the four reprocessing cycles, as seen in Figure 5B. The molecular weight of the feedstock was estimated to be Mn = 108,650 g/mol and Mw = 247,450 g/mol. During the first reprocessing cycle, both the Mn and Mw values of MR-1 were reduced by about 30%. In comparison, the Mn and Mw of SR-1 were reduced by 0.76% and 2.5%, respectively. These values are within the experimental error, and, therefore, it can be assumed that the solvent-based recycling technology does not induce significant degradation of the polymer chains, unlike the mechanical recycling approach. The molecular weight of mechanically recycled resins has a tendency to decrease, while solvent-based recycled resins exhibit an increasing trend. This is in good agreement with what has been observed with the MFI measurements. The molecular weight is the most probable preponderant factor inducing the MFI variations.

The MR pellet MFI observations can be explained by the degradation of PS induced by shear and elevated temperatures experienced during extrusion. This effect can be exacerbated owing to further catalysis from contaminants present in the resins [12]. Formulations designed for recycled resins can contain stabilizers and additives that prevent or counterbalance, to some extent, the degradation of the polymer. Depending on their efficiency, several cycles of reprocessing can be achieved for the recycled resins, yielding properties that are sufficient for further reprocessing/consumer use. A common strategy to overcome this molecular weight loss is to blend mechanically recycled resins with virgin resins possessing a lower MFI via masterbatch formulations. This approach permits maintenance of a constant MFI for the formulated resins. In comparison, because the SR pellets have a molecular weight close to that of the feedstock, they can be used directly as a replacement for their virgin counterpart. Alternatively, the SR pellets can be used in a blend with other sources of PS to achieve a target MFI. Furthermore, the solvent-based recycled resin exhibited an Mn increase, with an augmentation of 8.8% between the first and fourth cycle. This molecular weight rise could be explained by the removal of oligomers, or low-MW degraded polymer chain segments that are soluble in the selected solvents and can therefore be removed in the precipitation and washing stages. This phenomenon can be associated with the well-known polymer purification technique, removing monomers, dimers, and oligomers via re-precipitation. The reduction in the polydispersity (PDI) shown in Figure 5C for the SR samples is a good confirmation of this hypothesis.

**Figure 6:**
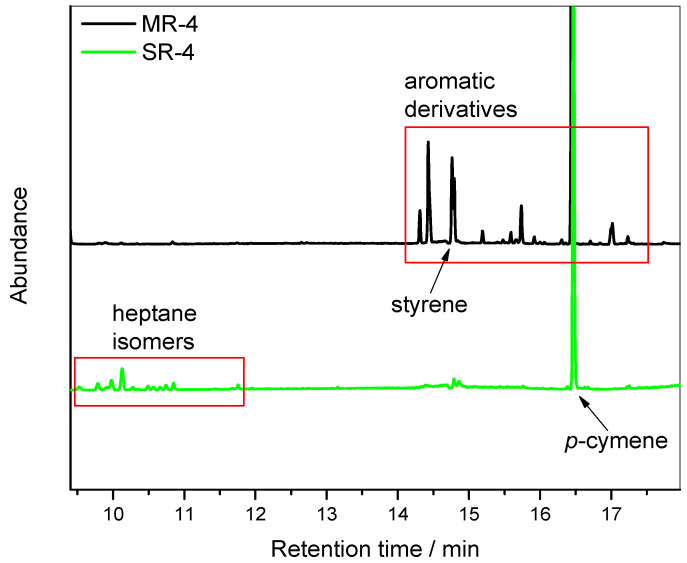
GC-MS chromatograms of the recycled resins after the 4th reprocessing cycle.

To uncover the impact of small molecules on the MFI results, a further investigation was conducted using GC-MS on the resulting recycled resins. Figure 6 exhibits the chromatograms of pellets from MR-4 and SR-4. The chromatogram from the MR-4 reveals the presence of several phenylic derivatives that are known products of the PS degradation process [22]. A list of assigned compounds from MS library can be found in Table S1 in the supplementary materials, along with the chromatograms and mass spectra. For instance, due to chain scission in an oxygen-containing environment, benzaldehyde and acetophenone were detected.

In comparison, the SR-4 sample exhibited only a few peaks, which mostly corresponded to residual solvents used in the recycling process. As specified in Figure 6, heptane isomers used as non-solvents were present at the concentration of 116 ppm. Similarly, the *p*-cymene, used as the primary solvent, could be found at the concentration of 1077 ppm. Due to the use of a pilot plant designed for research purpose, the devolatilization was not optimized (see Materials and Methods for more details). With an optimized devolatilization system, the remaining solvents could be further reduced.

In addition to the residual solvent content, the styrene monomer content was quantified. Styrene is always present in PS resins in trace amounts following the polymer synthesis pathway. Its concentration is regulated, particularly in applications involving food contact [24,25]. As seen in Table 2, the styrene monomer content remaining in the SR-4 resin was 75% lower than in the MR-4 sample. This can be explained by the phenomenology of the solvent-based recycling process. The styrene monomer is soluble in *p*-cymene and is thus removed during the precipitation and washing steps.

The decontamination efficiency of inorganic compounds from the PS can be obtained using X-ray fluorescence. Table 3 exhibits the results obtained from the starting material and all the recycled resins. The post-consumer starting material contains other materials in addition to PS that are additives related to the resin’s intrinsic formulation. It also contains contaminants that accumulate over their consumer life-time. With these in mind, the presence of bromine is often associated with flame retardants; zinc can be associated with lubricant additives such as zinc stearate; calcium can be related to calcium carbonate; and iron putatively comes from metal dust due to external contamination. The MR-based resins do not show any efficiency in removing the contaminants. The values are quite similar to those of the starting material but can be improved by adding filters into the extruder. However, filtering a polymer under melt conditions can be challenging due to the high viscosity and significant pressure drop during filtration. Therefore, particles below 500 µm, or 200 µm, depending on their nature, can be difficult to remove with this technology [26,27]. The observed difference between the starting material and each MR pellet shown in the Table 3 could be associated to the feedstock heterogeneity. Because of degradation, the residence time in the extruder must be minimized, thus the mixing is not optimized. Therefore, the sampling method has a significant impact on measurements. By averaging different samples, this impact tends to be minimized. The results from solvent-based recycling exhibit efficient decontamination. Chlorine, calcium, and iron are no longer present, with concentrations below their detection thresholds (Cl < 29.3 ppm; Ca < 19.8 ppm; Fe < 6.2 ppm). Less than 20 ppm of zinc remains in the same concentration for each resin. Similarly, bromine is present in very low concentrations of nearly 20 ppm [12]. Therefore, the remaining detected bromine could be associated with bromine that is present in the polystyrene backbone. Indeed, when processed with flame retardants, some bromines could be transferred onto the polystyrene chain. Because of the very low concentration of bromine sporadically transferred to the polymer chains, the PS chain that contains few atoms of bromine has a very similar solubility to the pure PS chain. Thus, the lightly brominated polymer chain cannot be removed easily through this process.

## 4. Conclusions

This investigation aimed to compare two different types of recycling technologies for EPS. Conventional mechanical recycling technology was compared with a newly developed physico-chemical recycling process with respect to the effects of each process on material Mn and Mw distribution and contaminants present in the recycled product, as well as the effects of these on melt viscosity and mechanical properties. As shown by the molecular weight decrease, the color, and the volatile chemicals released, mechanical recycling technology is limited by the quality of the feedstock. This approach can only use very clean feedstock, mostly white EPS, that has been pre-washed. This technology also necessitates additives such as stabilizers, radical scavengers, to be added to the formulation to maintain material properties over the numerous reprocessing cycles. These minimize degradation effects during extrusion.

Due to the very scarce amounts of very clean feedstock, other recycling solutions must be developed to respond to the challenge of recycling deeply contaminated PS feedstocks. The developed solvent-base technology described in this investigation demonstrates that it is possible to maintain the molecular weight of the PS feedstock while separating the degraded polymer chains. This process is also efficient in removing non-soluble and soluble contaminants such as dust and flame retardants. Moreover, the residual styrene monomer content is reduced by 75% via this process. These aspects are critical in developing recycled resins capable of substituting virgin materials from contaminated feedstock that cannot be achieved by simple surface pre-treatments such as water washing.

Similar to the concept of water washing, solvent-based recycling technology can be seen as a very efficient pre-treatment before extrusion. Herein, the key benefit offered by solvent-treatment is contaminant removal that is embedded into the polymeric network, i.e., at the molecular level, yielding highly purified resins. A perspective on this investigation would be comparing different pre-treatments with the same deeply contaminated feedstock to evaluate their efficiency versus their cost.

## Figures and Tables

**Table 1:** Light transmission and non-soluble contaminant content measurements for MR-4 and SR-4.

	MR-4	SR-4
Percentage of transmitted light (%)	50.1	96.7
Weight of remaining non-soluble (%)	0.82 ± 0.21	0.07 ± 0.03

**Table 2:** Styrene concentration comparison measured by GC-MS.

	MR-4	SR-4
Styrene concentration (ppm)	221 ± 14	55 ± 3

**Table 3:** X-ray fluorescence spectroscopy quantification of atom contaminants present in the PS materials tested.

	Cl (ppm)	Ca (ppm)	Fe (ppm)	Zn (ppm)	Br (ppm)
Feedstock *	99 ± 35	793 ± 75	350 ± 46	110 ± 20	100 ± 10
MR-1	-	381 ± 59	63 ± 41	57 ± 5	25 ± 3
MR-2	40 ± 31	535 ± 66	303 ± 50	75 ± 6	137 ± 6
MR-3	34 ± 33	483 ± 63	272 ± 49	69 ± 6	117 ± 6
MR-4	142 ± 43	534 ± 66	328 ± 51	67 ± 6	119 ± 6
SR-1	-	-	-	15 ± 3	16 ± 2
SR-2	-	-	-	12 ± 3	22 ± 3
SR-3	-	-	-	15 ± 3	23 ± 3
SR-4	-	-	-	14 ± 3	27 ± 3

* Due to the expanded nature of the EPS starting material, XRF could not be performed directly on the EPS. Therefore, the measurement was conducted on a solution of 20 wt.% of feedstock in *p*-cymene. The exhibited results were extrapolated for a 100% PS matrix.

## Data Availability

Data are contained within the article.

## Supplementary Materials

The following supporting information can be downloaded at: https://www.mdpi.com/article/10.3390/polym15244714/s1, Figure S1: Chromatograms of the recycled materials; Table S1: Peak analysis from the chromatogram of MR-4.; Figure S2: MS spectra corresponding to the chromatogram peak at the specified retention time.
