# Supplementary material for: Recyclability of Post-Consumer Polystyrene at Pilot Scale: Comparison of Mechanical and Solvent-Based Recycling Approaches
Source: Polymers (Basel). 2023 Dec 15;15(24):4714. doi: 10.3390/polym15244714 (PMC10748170; doi:10.3390/polym15244714)
Supplement: Supplementary file 1 [file polymers-15-04714-s001.zip › polymers-2699794-supplementary.pdf]

Supporting Information

-

Recyclability of Post-Consumer Polystyrene at Pilot Scale: Mechanical versus Solvent-based Recycling Approaches

Jean-Mathieu Pin[1]*, Iman Soltani[2], Keny Negrier[1], Patrick Lee[2]*

[1] Polystyvert Inc., 9350 Rue de l'Innovation, Anjou, QC H1J 2X9, Canada
[2] Multifunctional Composites Manufacturing Laboratory (MCML), Department of Mechanical and Industrial Engineering, University of Toronto, 5 King's College Road, Toronto M5S 3G8, Canada

* Corresponding authors: jmpin@polystyvert.com; patricklee@mie.utoronto.ca

I) <u>GPC analysis</u>

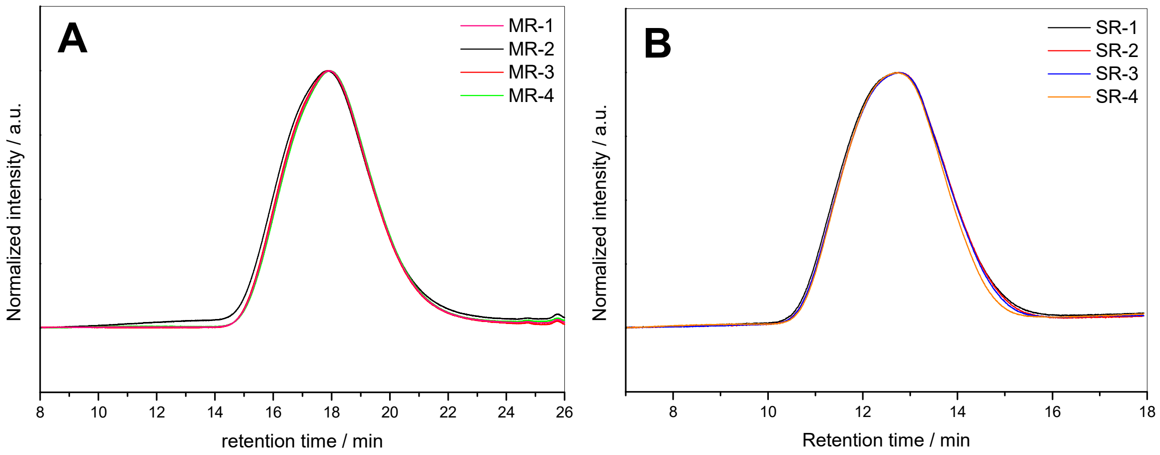

Figure S1. Chromatograms of the recycled materials

II) GC-MS analysis

Table S1. Peak analysis from the chromatogram of MR-4.

Retention time / min	NIST MS library assignation
14.32	ethylbenzene
14.45	*p*-xylene
14.76	styrene
15.20	cumene
15.59	*n*-propylbenzene
15.74	benzaldehyde
16.43	p-cymene
17.02	acetophenone

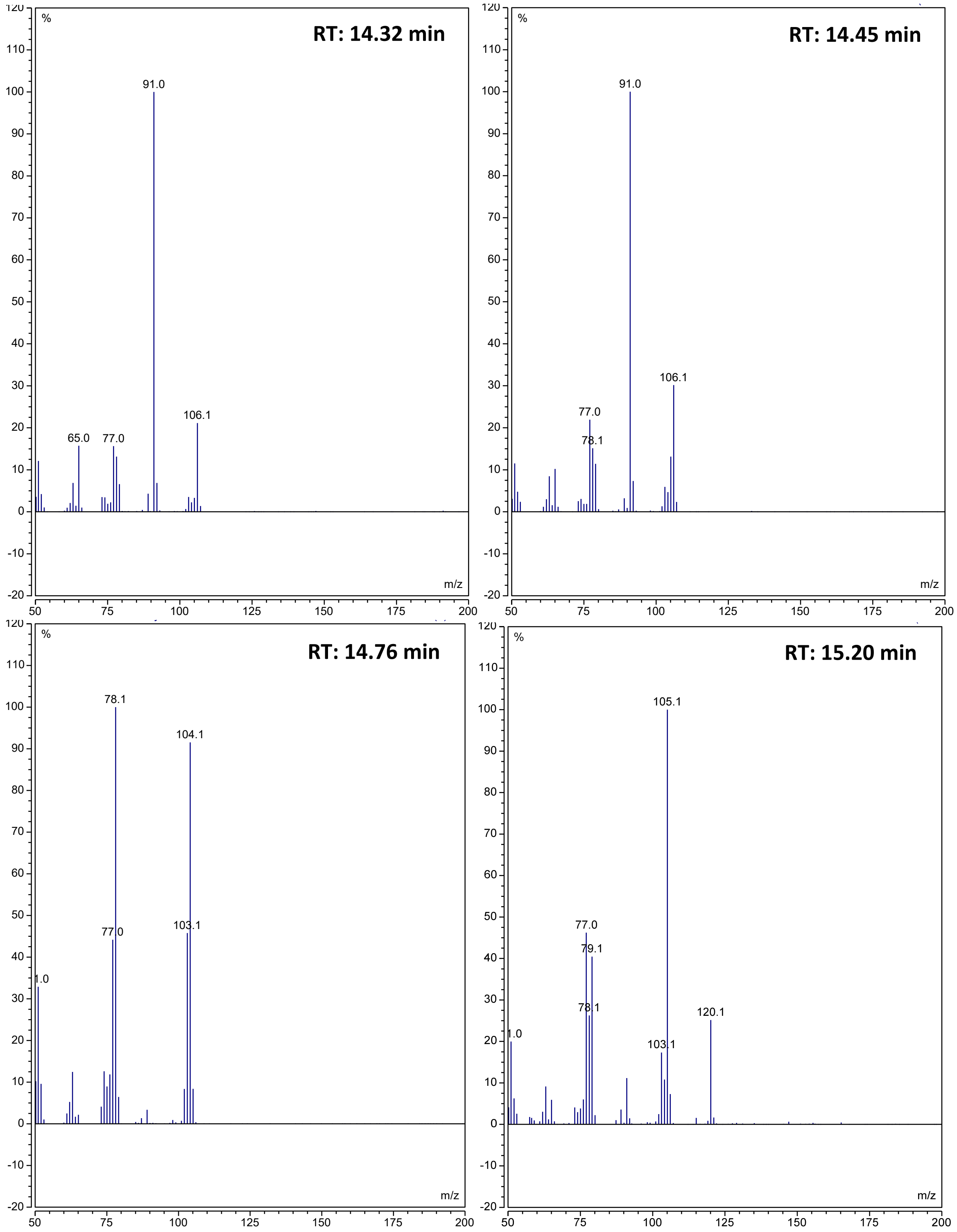
RT: 14.32 min
%
91.0
106.1
65.0
77.0
m/z
RT: 14.45 min
%
91.0
106.1
77.0
78.1
m/z
RT: 14.76 min
%
78.1
104.1
77.0
103.1
1.0
m/z
RT: 15.20 min
%
105.1
77.0
79.1
78.1
120.1
103.1
1.0
m/z

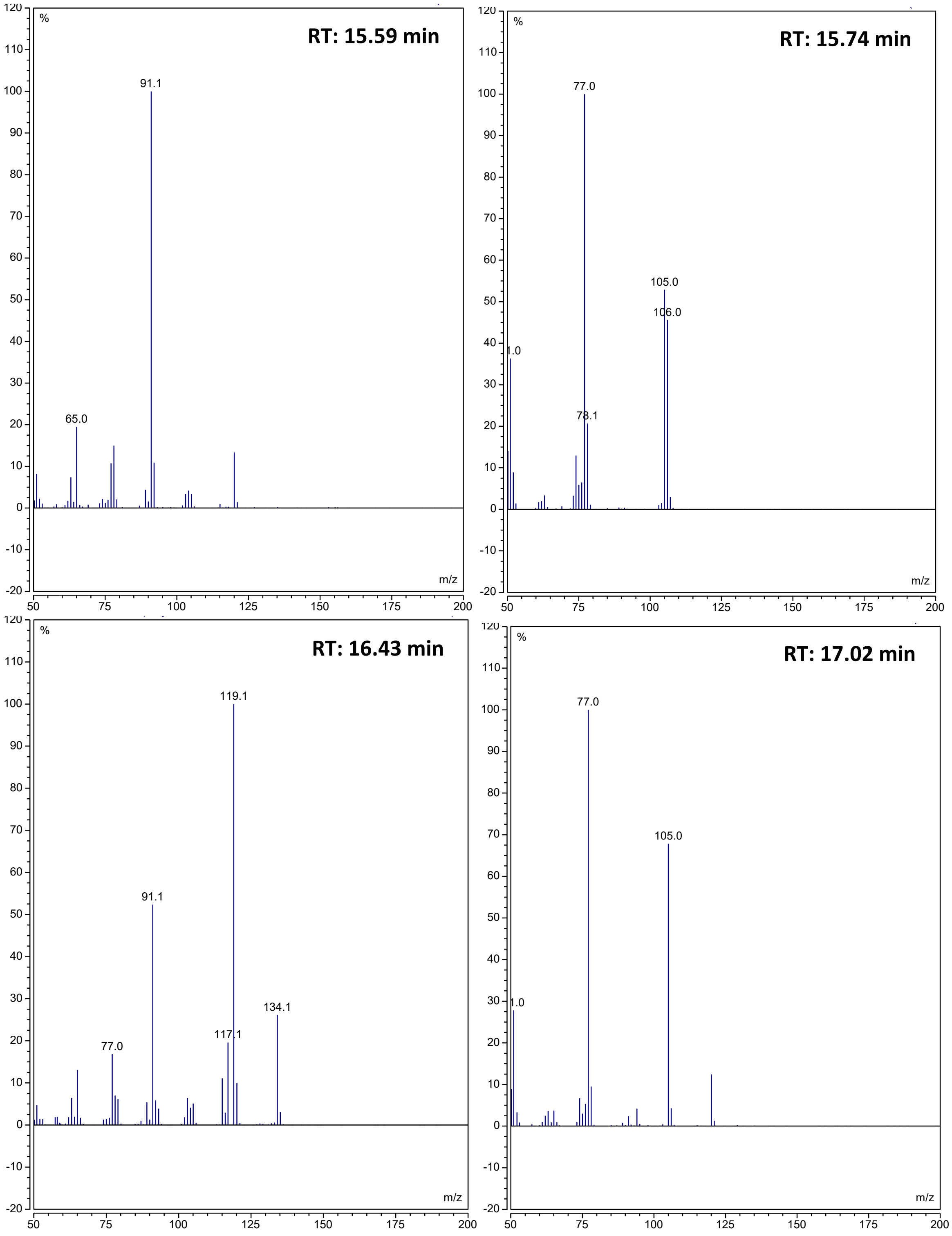

Figure S2. MS spectra corresponding to the chromatogram peak at the specified retention time.
